# A Novel Grouting Diffusion Monitoring System Based on ZigBee Wireless Sensor Network

**DOI:** 10.3390/s25092693

**Published:** 2025-04-24

**Authors:** Xiangpeng Wang, Tingkai Wang, Jinyu Gao, Meng Yang, Fanqiang Lin, Yong Jia

**Affiliations:** 1 Key Laboratory of Earth Exploration and Information Technology of Ministry of Education, Chengdu University of Technology, Chengdu 610059, China; xiangpengwang@cdut.edu.cn; 2 College of Geophysics, Chengdu University of Technology, Chengdu 610059, China; 3 College of Mechanical and Electrical Engineering, Chengdu University of Technology, Chengdu 610059, China; 2023021203@stu.cdut.edu.cn (J.G.); cdut.daoshan@gmail.com (M.Y.); linfq@cdut.edu.cn (F.L.); jiayong2014@cdut.edu.cn (Y.J.)

**Keywords:** grouting effectiveness, diffusion monitoring, ZigBee, non-destructive, apparent resistivity

## Abstract

Grouting technology is widely used in construction and civil engineering, where evaluating grouting effectiveness is crucial due to the uncertainty of subsurface conditions. Existing methods face drawbacks such as destructiveness, high cost, poor durability, and limited data collection. To address these issues, this paper proposes a novel wireless real-time monitoring system based on a ZigBee sensor network framework. The sensor system integrates a direct current method in geophysics with apparent resistivity measurement to assess grouting effectiveness in real time. It consists of multichannel data acquisition units with electrodes for sensing underground currents and a user control unit for centralized management and data processing. A system acquisition performance test confirmed that the differential input channel’s equivalent input noise of the ADC was only 175 μV and 188 μV, and the average error of the captured sine wave data was 4.51 mV and 4.19 mV, ensuring the voltage measurement accuracy of the data acquisition units. Stability testing of the equipment in road and construction environments showed an average RSD of 2.86% and 2.92%, respectively, indicating good stability of the measurements. ZigBee network performance tests in three simulated environments and a field test showed that the packet loss rate (PLR) was less than 2% from 0 to 50 m, ensuring network communication in grouting project scenarios. On-site experiments demonstrate that the system can simultaneously monitor multiple profiles and perform inversions in the grouting area, which can be assembled into 3D inversion images for evaluating grout diffusion, offering valuable insights for optimizing construction operations, and enhancing grouting efficiency.

## 1. Introduction

Grouting technology is widely used in the fields of building construction and civil engineering to address challenges such as waterproofing, soft rock reinforcement, and backfilling of mined-out areas [1,2,3,4]. For instance, in tunnel engineering, the grouting method is generally used to solve the engineering and technical problems of water-related distresses such as water leakage and water inrush, which affect the normal operation of tunnels [5]. For the formation of goafs caused by mining activities, the grouting reinforcement method is a commonly used remedial approach for goafs [6]. The fundamental principle of grouting involves injecting grout mixtures into soil or rock strata under controlled pressure, allowing the grout to permeate, diffuse, and fill pores, fractures, and voids, thereby improving the physical and mechanical properties of these structures [7,8]. Through grouting reinforcement, the overall stability and self-supporting capacity of the structure are significantly enhanced.

Currently, grouting technology demonstrates significant application effectiveness across various fields [9,10,11]. However, the accurate evaluation of grouting effectiveness remains a focal issue of considerable attention. The existing evaluation methods mainly include the analytical method, the inspection hole test method, excavation sampling, and the geophysical exploration method. The analytical method is based on a theoretical model of the grouting diffusion mechanism and combined with numerical simulation to assess the effectiveness of grouting [12]. This method provides important theoretical support for grouting projects, but its accuracy relies on the collection of field data for calibration and validation. The inspection hole test method and excavation sampling directly observe the grout diffusion through drilling or excavation and analyze the density and uniformity of the grout by sampling to verify whether the grouting has achieved the desired effect. Although these direct methods offer intuitive assessment results, they are generally time-consuming, operationally complex, and costly [13,14], leading to limited applications in the real world.

Therefore, this study is dedicated to developing a highly efficient, non-intrusive, and real-time grouting evaluation system, proposing a novel distributed real-time sensor system for monitoring grout diffusion based on a ZigBee-endowed framework. This system incorporates the direct current (DC) method from geophysical methods and combines it with apparent resistivity measurements, innovatively applying it to grouting monitoring. Specifically, electrodes are arranged on the ground surface of the grouting area, where one set of electrodes periodically applies direct current while another set measures voltage. By calculating the apparent resistivity and performing inversion processing, the system enables real-time monitoring of the diffusion and dynamic evolution of grout in the subsurface. It is worth noting that the proposed system innovatively achieves non-invasive real-time monitoring of grout diffusion through the integrated application of a multi-channel electrode array, ZigBee wireless networking, direct current resistivity measurement, and apparent resistivity inversion techniques. Specifically, the system’s data acquisition unit is equipped with up to 60 channels and employs time-division multiplexing technology, allowing simultaneous observation of multiple profiles and combining into intuitive three-dimensional inversion images. This design effectively reduces measurement errors caused by frequent electrode insertion and removal, ensuring measurement accuracy and spatial resolution. Meanwhile, the system utilizes a ZigBee wireless network to enable real-time communication between the operation control unit and multiple data acquisition units, simplifying wiring design and enhancing the system’s scalability and sustainability for long-term monitoring. This makes the system more adaptable to grouting projects of varying scales. On-site testing experiments demonstrate that the proposed system can perform real-time three-dimensional inversion of the grouting process and effectively monitor grouting effectiveness, providing reliable technical support for grouting engineering.

To address the limitations of existing grouting evaluation methods and to improve efficiency, real-time performance, and non-intrusiveness of monitoring, this paper proposes a novel distributed monitoring system based on the ZigBee wireless sensor network and direct current method. Section 2 reviews related studies on grouting effectiveness assessment techniques and the current status of wireless communication technologies applied in engineering monitoring. Section 3 describes the measurement principles of the proposed system and presents its overall structural composition. Section 4 details the hardware architecture and key circuits of the data collection unit, while Section 5 presents the software implementation and communication workflow. Section 6 presents experimental validation and performance tests in both laboratory and field tests. Section 7 discusses the advantages, limitations, and potential improvements of the system. Finally, Section 8 summarizes the key findings and provides prospects for future work.

## 2. Related Work

### 2.1. Current Research on Grouting Effectiveness Evaluation Methods

Current methods for assessing grouting effectiveness can be primarily categorized into two main groups: invasive and non-invasive [15]. Invasive methods mainly include the inspection hole method and the excavation sampling method [16,17]. The inspection hole method assesses the density and uniformity of the grouted area directly by drilling holes and extracting samples, and the excavation sampling method observes and measures the grouting effects directly through physical excavation of the grouted area.

In prior studies, the Measurement While Drilling (MWD) technology has been employed to investigate drill monitoring for rock mass grouting. Although it demonstrates strong predictive capabilities for rock mass quality and grouting consumption, it is costly, notably destructive, and the data interpretation is complex [18]. During the construction of deep roadways, excavation and borehole drilling methods have been introduced to assess the reinforcement effects of grouting. It has been concluded that grouting can significantly improve the integrity and stability of fractured rock masses surrounding deep roadways, thus providing a technological guarantee for the safe production of deep coal mines [19]. Wang et al. [17] evaluated the actual grouting effect using the excavation sampling method, verifying the rationality and feasibility of the proposed expansion theory. These methods and techniques have provided detailed local information and reference analyses for the study of grouting effectiveness. However, they are characterized by strong invasiveness and destructiveness, causing certain degrees of damage to the grouted areas. They also face challenges in real-time performance, energy consumption, and data processing efficiency.

In contrast, non-invasive methods assess grouting effectiveness without causing significant damage to the grouted area. As these methods provide comprehensive evaluations while preserving structural integrity, they are increasingly favored by researchers. Geophysical methods, such as seismic waves [20], ground-penetrating radar (GPR), and resistivity imaging [21], are widely used non-invasive techniques for evaluating grouting effectiveness [22,23,24,25]. These methods provide valuable data by detecting changes in physical properties or the elastic mechanical distribution of subterranean structures without direct intervention. However, GPR has limitations in detection depth. Although reducing the frequency can increase the detection depth, it leads to a decrease in resolution [26]. Additionally, it is easily affected by high-conductivity and inhomogeneous conditions [27]. It also incurs high equipment and operation costs for large-scale projects. Among them, the direct current (DC) method stands out for its non-invasive nature and ease of deployment and data interpretation. It works by placing pairs of electrodes on the ground surface, applying a DC current to the subsurface medium, and measuring the potential difference between the electrodes to calculate the resistivity distribution of different subsurface locations. Due to these advantages, the DC method is widely applied in various fields. For instance, Li et al. [24] utilized this method to detect faults in the basalt-covered area of Changbai Mountain. They successfully performed inversion analysis to delineate the distribution patterns of the northeastern branch of the Yalu River Fault Zone extending from the Songhua River’s banks towards the northeast of Songjianghe Town. DONG and WANG [28] applied the forward modeling theory of the DC resistivity method to establish models of complete strata, high-resistivity, and low-resistivity formations.

Despite the significant advancements of the DC resistivity method as a non-invasive technique, several challenges persist. These include enhancing resolution and detection depth, as well as integrating the method into a unified monitoring system. Building upon prior research in DC resistivity and following meticulous evaluation, this paper proposes a novel wireless distributed real-time monitoring system based on the DC resistivity method for evaluating grouting effectiveness.

### 2.2. Current Status of Wireless Sensor Network Communication

Wireless Sensor Networks (WSNs) are distributed sensing networks that transmit sensor data and information via wireless communication technologies. Common wireless communication technologies include WiFi, Bluetooth, LoRa, and ZigBee. These technologies have been widely applied across various scenarios and fields due to their unique performance characteristics [29,30,31,32]. For example, a smart keychain based on the STM32 microcontroller and Bluetooth positioning technology has been developed. This keychain employs a Bluetooth fingerprint-based Received Signal Strength Indication (RSSI) localization algorithm to achieve precise localization within indoor or short-range environments [33]. Some studies have proposed a multi-hop network architecture based on LoRa technology, applying it to a distributed measurement system [34] and an earthquake early warning system [35]. Through the multi-hop forwarding mechanism, they have achieved low-power, long-range communication while significantly enhancing the system’s reliability and real-time performance. Additionally, a WiFi-based gesture recognition method has been proposed, which extracts the Doppler shift component from the channel state information of WiFi signals to address the unclear mapping between statistical features and specific gesture actions in wireless gesture recognition methods [36]. However, no related work has been found that applies LoRa, Wi-Fi, or Bluetooth technologies in the field of grouting diffusion monitoring.

Drawing on studies of wireless communication technologies [37,38,39,40,41], we compared the performance metrics of Bluetooth, ZigBee, Wi-Fi, LoRa, and NB-IoT in Table 1. NB-IoT is not a WSN, but like LoRa, it belongs to LPWAN technologies and is thus considered in the comparison of wireless communication technologies. Despite their widespread use, each of these technologies has certain limitations [42,43,44]. For example, Wi-Fi suffers from high power consumption, limited coverage, and high cost. Bluetooth is suitable for short-range transmission but can connect only a limited number of devices. LoRa has low data transmission rates (<50 kbps) and high latency, making it more suitable for sporadic reporting rather than continuous real-time monitoring [38]. NB-IoT relies on mobile network infrastructure and has a relatively high deployment cost, making it unsuitable for remote areas without mobile connectivity [38,45]. In contrast, ZigBee can be configured with a mesh network topology, where each node can communicate with its neighboring nodes, enabling large-scale deployment [46]. Additionally, ZigBee offers high reliability and low power consumption, ensuring efficient data processing, transmission, and real-time performance. Although its communication range is relatively short, ZigBee can meet the requirements of most grouting projects through signal relaying. ZigBee has also been widely applied in other engineering fields. For example, a data acquisition substation for mining equipment was designed using ZigBee wireless sensor network technology and an STM32 embedded system, achieving reliable and efficient data collection through large-scale deployment [47]. Similarly, a ZigBee-based wireless monitoring system was developed to monitor environmental parameters in open-pit and underground mining operations [41]. The real-time monitoring capability of this system can effectively prevent safety accidents in mines, highlighting its significant practical application value. Drawing on these characteristics and previous studies, this study employs ZigBee technology to develop a grouting monitoring system, establishing a distributed grouting diffusion monitoring network. This not only expands the application scope of WSN technology but also overcomes the limitations of previous studies that relied heavily on wired systems or manual measurements.

## 3. Methodology

**Figure 1:**
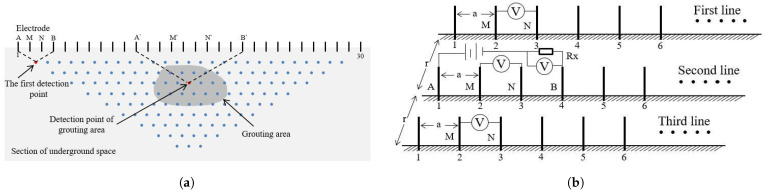
Schematic diagram of multichannel acquisition using direct current electric method. (**a**) Measurement principle. (**b**) Schematic diagram of parallel Wenner device. In (**a**), A′, B′, M′, and N′ represent the electrode utilized for a subsequent data acquisition at a different location from the first acquisition.

During the grouting process, the electrical properties of the slurry change the resistivity of the grouted area. This system utilizes the direct current method from geophysical techniques, using a specific array of electrodes placed on the surface, eliminating the need to bury any equipment. By measuring the voltage change during grouting, this system calculates the apparent resistivity and performs an inversion to evaluate the grouting diffusion. Although soil moisture content affects the background apparent resistivity values of the stratum, the diffusion of grout can still be effectively monitored by comparing the relative changes in apparent resistivity before and after grouting. The measurement principle is shown in Figure 1a, which indicates a profile measurement diagram of a measurement line. Electrodes are placed on the ground in the measurement area, some of which are connected to a power source for supplying electricity, while others are used to collect voltage data transmitted through the subsurface medium. By selecting different electrodes as power supply and measurement electrodes and adjusting the spacing between them, the depth and location of the detection point can be changed.

The monitoring system in this study uses a Wenner array for data acquisition, as shown in Figure 1b, where electrodes *A* and *B* are used as power supply while *M* and *N* are used as measurement electrodes. The distance between the measurement lines is *r* and the electrode spacing is *a*. In this system, all electrodes are deployed on the ground surface without burial. The spacing parameters (a and r) are adjustable based on the required measurement depth and project scale. For example, larger spacings (e.g., a = 1–10 m) enable deeper measurements, while smaller spacings (e.g., a = 0.1–0.5 m) provide higher resolution for shallow regions. Therefore, the electrode spacing and line spacing should be flexibly selected according to the specific requirements of different grouting applications. When *A* and *B* are discharged, a current passes through the subsurface medium and generates an electric field. The distribution pattern of the current field reflects the geologic structure and electrical inhomogeneity of the region. After the voltage difference $\Delta U_{MN}$ is measured between *M* and *N*, the equivalent apparent resistivity $\rho_{s}^{\mathrm{AB}}$ beneath the midpoints of electrodes *A* and *B* can be calculated by Equation (1):

(1) $$\rho_{s}^{\mathrm{AB}}=K_{AB}\times \frac{\Delta U_{MN}}{I}$$

In the formula, $K_{AB}$ represents the system’s device coefficient, which can be calculated using Equation (2):

(2) $$K_{AB}=\pi \cdot \frac{d_{\mathrm{AM}}\cdot d_{\mathrm{AN}}}{d_{\mathrm{MN}}}$$

In the formula, $d_{AM}$, $d_{AN}$, $d_{MN}$ represent the distances between electrodes *A* and *M*, *A* and *N*, *M* and *N*, respectively. The device coefficient is calculated as follows:

(3) $$K=\frac{2\pi }{\left(\frac{1}{d_{AM}}-\frac{1}{d_{AN}}-\frac{1}{d_{BM}}+\frac{1}{d_{BN}}\right)}$$

The above three equations are the fundamental formulas for the Wenner array [48]. For the Wenner device used in this monitoring system, $d_{AM}$ and $d_{AN}$ are equal, so Equation (2) is obtained. The parallel measurement of the Wenner device can be derived from Equation (3) to Equation (4):

(4) $$K=\frac{2\pi }{\left(\frac{1}{\sqrt[{d}]{AM^{2}+r^{2}}}-\frac{1}{\sqrt[{d}]{AN^{2}+r^{2}}}-\frac{1}{\sqrt[{d}]{BM^{2}+r^{2}}}+\frac{1}{\sqrt[{d}]{BN^{2}+r^{2}}}\right)}$$

In the formula, AM/AN is the interval distance between parallel measurement *A* electrode and *M* electrode/*N* electrode, BM/BN is the interval distance between *B* electrode and *M* electrode/*N* electrode, and *r* is the interval distance between measurement lines. Therefore, the formula can be simplified as

(5) $$K=\pi \cdot \frac{\sqrt{AM^{2}+r^{2}}\cdot \sqrt{AN^{2}+r^{2}}}{\sqrt{AM^{2}+r^{2}}-\sqrt{AN^{2}+r^{2}}}$$

By arranging multiple measurement lines in the grouting area and collecting measurement data from multiple profiles simultaneously, it is able to invert the 3D apparent resistivity results in real time to detect the diffusion of the slurry and the grouting effect timely. In view of this, the monitoring system studied in this paper is designed to be able to monitor multiple measurement lines in parallel and has expandability to meet the monitoring of grouting areas of different scales.

**Figure 2:**
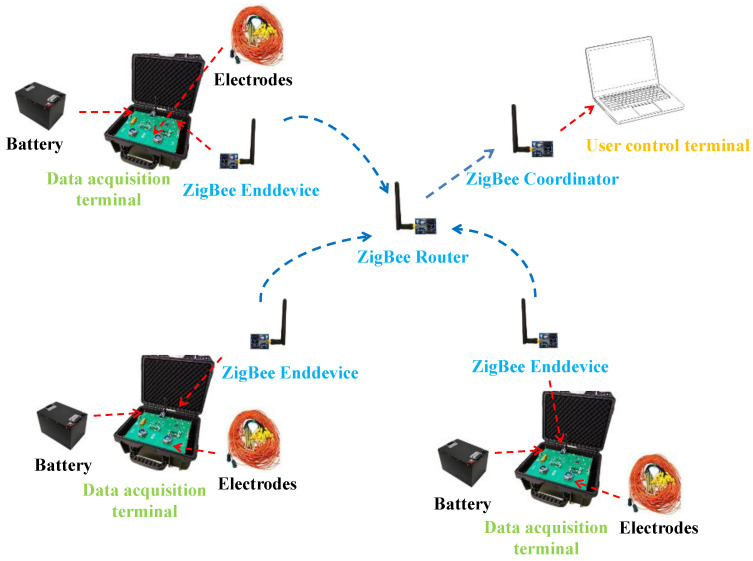
Composition structure of the monitoring system.

The structural composition is shown in Figure 2. The system is divided into three parts: user control unit, ZigBee wireless communication network, and data acquisition unit. The user control unit contains a computer and a ZigBee module, and a QT-based monitoring software is designed to control the data acquisition unit and receive the acquired data in real time. The ZigBee wireless communication network comprises a number of CC2530-based ZigBee modules for transmitting data and commands between the data acquisition unit and the computer. The user control unit acts as a coordinator node and the data acquisition unit works as an end-device node. The data acquisition unit consists of a data acquisition board, an electrode control board, and a ZigBee module for implementing measurement acquisition of the grouted area. Each data acquisition unit utilizes up to 60 electrode channels. It allows multiple data acquisition units to be controlled in real time by operating the control unit, or each data acquisition unit can also be operated independently.

## 4. Hardware Design

**Figure 3:**
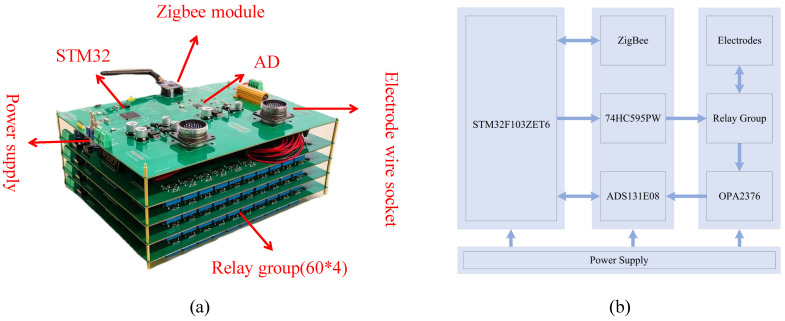
Schematic and block diagram of the data collection unit circuit board. (**a**) Data collection unit PCB. (**b**) Block diagram of data collection unit structure.

As Figure 3a illustrates the structure and design of the data acquisition unit, the data acquisition unit consists of a stack of five circuit boards, including a control board and four relay boards, which are connected to each other using wires to enable the control board to operate the relays on the four relay boards. Figure 3b shows the main hardware of the data acquisition unit and its relations. The unit utilizes STMicroelectronics’ (Geneva, Switzerland) ARM 32-bit Cortex™-M3 architecture STM32F103ZET6 chip as the microcontroller unit (MCU). The TI CC2530-based ZigBee module, developed by Texas Instruments (Dallas, TX, USA), integrates an RF transceiver and an 8051 microcontroller core running on the ZigBee2007 network protocol stack. The module communicates data with the processing unit through a serial interface. To control the 60 relays of the 4 relay boards using the MCU’s IO interfaces, the unit employs 32 74HC595PW serial-to-parallel chips, developed by Nexperia (Nijmegen, The Netherlands), to process the 60 × 4-bit serial data outputs. The 60-bit serial output from each IO controls 60 relays on a single relay board, enabling management of 240 relays on all four boards. The acquisition voltage is processed using an OPA2376 operational amplifier and converted to a digital signal using a 24-bit ADS131E08, both developed by Texas Instruments (Dallas, TX, USA). A 12 V lithium battery powers its chip while a 48 V battery provides the discharge voltage.

### 4.1. Relay Control Circuit

**Figure 4:**
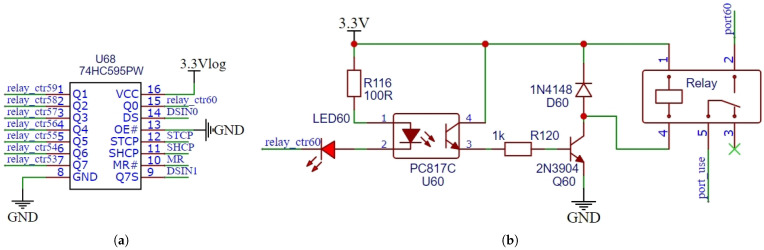
Principle diagram of the electrode switching control circuit. (**a**) 74HC595PW serial to parallel circuit. (**b**) Relay control circuit.

The STCP, SHCP, MR, and DS pins of the 74HC595PW chip are connected to the MCU as shown in Figure 4a. The MCU sends serial data to the chip through the DS pin and locks it, then outputs it through the Q0-Q7 pins. The Q7S pin of the 74HC595PW is connected to the DS pin of the next 74HC595PW chip in order to realize a cascade connection of eight 74HC595PW chips. In this way, 60 relays can be managed by the control circuit shown in Figure 4b. Similarly, the MCU is able to control a total of 240 relays on four relay control boards. In Figure 4b, the common Terminals 5 of all the relays on the relay boards are connected to each other and the normally closed Terminals 2 are connected to the electrodes. The four relay boards of each data acquisition unit correspond, respectively, to the selection of *A*, *N*, *M*, and *B* electrodes. Specifically, each of the 60 relays of each relay board correlates to an electrode, and activating a relay selects its corresponding electrode as one of the four used electrodes. When the relay is disabled, the normally closed Terminal 2 is disconnected from the common terminal and makes no electrical connection. The data acquisition unit can be connected to 60 electrodes, referred to as 60 channels. During data acquisition, four electrodes are used for each measurement, where A and B are used for discharge and M and N are used for voltage signal acquisition. The system polls electrodes 1 to 60 in sequence. To prevent cross-talk issues, the system employs mechanical relays and optocouplers for polling the electrodes. Additionally, during channel switching, reverse discharge and software delays are used to reduce residual charges underground. These two methods help to minimize residual voltage and switching leakage current, further reducing asynchronous acquisition cross-talk.

### 4.2. Analog-to-Digital Conversion Circuit

**Figure 5:**
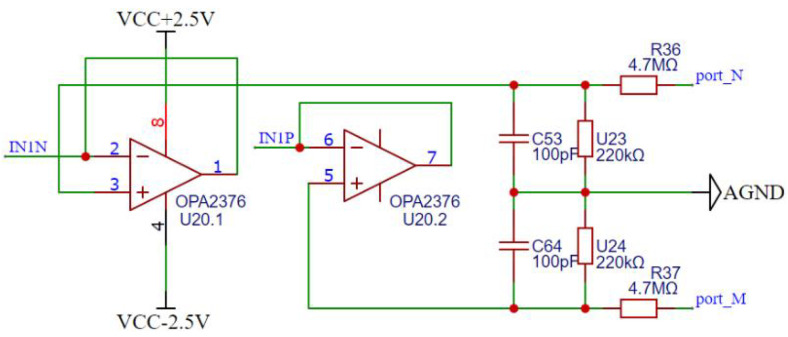
Differential input attenuation circuit of MN electrode.

As shown in Figure 5, a high-precision voltage attenuation circuit consisting of resistors and an OPA2376 operational amplifier is demonstrated to improve the voltage acquisition capability and input impedance of the data acquisition unit prior to A/D conversion of the voltage. The circuit reduces the differential voltage between electrodes *M* and *N* by a factor of 22.36, enabling the unit to handle voltages as high as 55.9 V. In addition, a voltage divider circuit was designed to measure the battery voltage to calculate the battery output current. A 1-ohm sampling resistor $R_{x}$ forms a differential input attenuation circuit with a two-fold attenuation factor. The voltages of the measurement electrodes, the battery, and the sampling resistor are attenuated through the differential input circuit and are fed to the ADS131E08 chip for voltage conversion. The sampling rate of the ADS131E08 chip is set to 1 kHz. The microprocessor retrieves and calculates the voltage difference ΔV between electrodes *M* and *N*, and the output current *I* of the MN circuit and the battery pack through SIP communication.

## 5. Software Design

**Figure 6:**
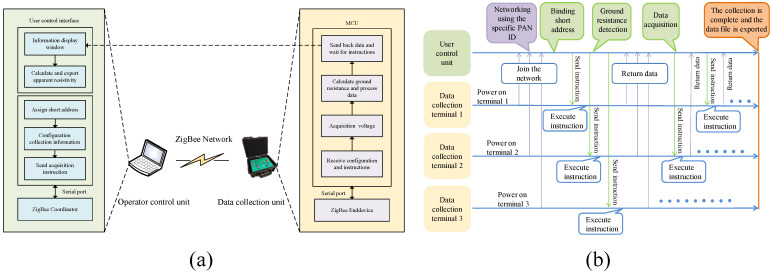
Architecture and workflow design of software system. (**a**) System architecture diagram. (**b**) Workflow diagram of real-time monitoring system.

Figure 6 represents the functional architecture and interaction flow of the system at the software level. Figure 6a shows the software functional architecture of the proposed system according to the three modules: user control unit, data acquisition unit, and ZigBee network. In the control unit, a user control interface is designed, which contains an information display window for monitoring the acquisition data and information, and an acquisition setting area that allows the user to configure data acquisition parameters and send acquisition commands. In addition, the software assigns unique sequential numbers to the data acquisition units in joining the network to facilitate the differentiation of different data acquisition units. As shown in Figure 6b, by binding the sequence number to the short address of the acquisition unit, it is convenient for the user to manage the data acquisition unit through the sequence number. The ZigBee module program is responsible for creating and configuring the ZigBee network (e.g., channels and PANIDs), managing the multiple ZigBee nodes, and realizing the data transmission from the acquisition unit to the user’s control unit. The acquisition unit program performs acquisition operations, calculating and processing the acquired data based on the configured parameters. According to Figure 6b, the steps for the user to operate the system are as follows. First, the user control unit and the acquisition unit are turned on to establish the ZigBee network. The acquisition unit is recognized by the control interface after successful network connection and is assigned a number. Second, the acquisition settings are configured in the interface, the device number is entered to select the specified acquisition unit, and commands and settings are sent. Third, the acquisition unit collects and processes the data according to the configuration and transmits it back to the user control interface, where the user monitors the data in the information display window. Finally, the apparent resistivity can be calculated through the user control interface and exported for inverse analysis.

In the ZigBee network, the ZigBee module of the operation control unit acts as a coordinator node and the ZigBee module of the data acquisition unit acts as an end device node. The network uses a mesh network topology. All ZigBee nodes are configured with the same PANID, channel, and network key. Selection of a specific PANID helps to avoid interference from other ZigBee networks, and selection of an appropriate channel reduces jamming from other 2.4 GHz ISM band devices (e.g., Wi-Fi and Bluetooth), thus ensuring network stability. Network keys are used for data encryption and decryption to protect broadcast communications and any network layer communications between ZigBee devices [49]. The encryption method uses AES-128 encryption to protect the confidentiality of the data [50]. These methods are employed to enhance the reliability of the network, minimize external interference, and ensure data security. For node management, the ZigBee coordinator establishes the network and assigns a unique 16-bit network address to each newly joined device. The router node (if required) maintains the network topology and ensures stable connections between nodes. In case of node failure, the ZigBee network performs a self-healing function to update the communication link and maintain stability.

**Figure 7:**
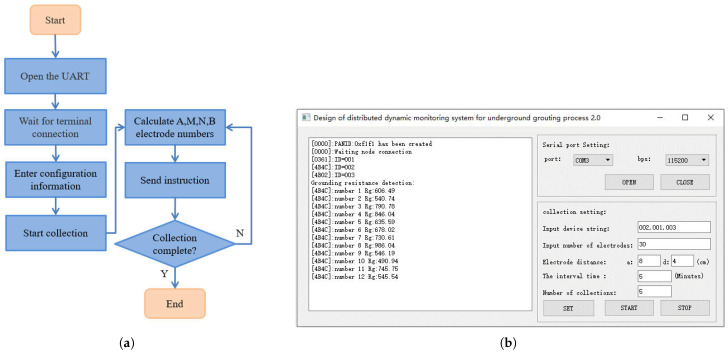
Schematic diagram of real-time acquisition using direct current electric method. (**a**) The operation process of unit control. (**b**) User operation interface.

The user control interface receives information transmissions from the ZigBee coordinator via the serial port and enables the operation and control of the data acquisition units. As shown in Figure 7b, the short address and assigned number of the newly added device are displayed in the information display window, allowing the user to ensure that all data acquisition units are correctly connected to the ZigBee network. Depending on the actual measurement requirements, the user is required to manually enter the acquisition parameter information. The first numbered device acts as the primary unit for both discharge and data collection, while the subsequent devices function solely as secondary units for collection.

Prior to initiating data collection, conducting a ground resistance test on each electrode is crucial to ensure proper contact with the ground. The operator control unit issues command signals to the data collection unit, progressing through the electrode numbers in ascending order. For each command, it activates two adjacent electrodes and collects voltage data at either unit of the sampling resistor $R_{x}$. Upon executing the command, the data collection unit relays back the measured voltage values. The operational workflow of the data collection unit is depicted in Figure 7. In a scenario where two adjacent electrodes function as *A* and *B* during discharge, the power supply, electrode *A*, the ground, electrode *B*, and the sampling resistor $R_{x}$ constitute a circuit. $R_{x}$ is linked in line with the ground’s equivalent resistance. The grounding resistance $R_{g}$ is calculated using Equation (6) based on voltage divider measurements.

(6) $$R_{g}=\frac{\left(V-U_{Rx}\right)\cdot R_{x}}{U_{Rx}}$$

In the formula, *V* is the voltage of the battery pack, $V_{Rx}$ is the voltage across the sampling resistor $R_{x}$.

Upon the completion of the grounding resistance testing, the data collection process can commence. The user control unit sequentially sends control commands to each unit device, guided by the predetermined electrode numbering. During multi-machine real-time acquisition, the secondary units do not require discharging, hence the values corresponding to electrodes *A* and *B* are set to zero in each instance.

For example, during a collection process in Figure 6b, assuming that Data Collection Unit 2 is the host, Units 1 and 3 are secondary, and the calculated values of *A*, *M*, *N*, and *B* correspond to 1, 2, 3, and 4, the following data interaction is performed:

1. The user controls the unit to send instructions *A*, *M*, *N*, and *B* of 1, 2, 3, 4 to the Data Acquisition Unit 2.
2. Data Collection Unit 2 carries out commands to manage the relay assigning electrodes *A*, *M*, *N* and *B* to Positions 1 through 4. It collects voltage $V_{MN2}$ between the power supply voltage $V_{B2}$ and MN and samples the resistance voltage $V_{Rx2}$ before sending it to the operator control unit.
3. The user control unit sends instructions with *A*, *M*, *N*, and *B* values of 0, 2, 3, and 0 to Data Collection Units 1 and 3 in sequence.
4. Data Acquisition Units 1 and 3 execute instructions to control the relay to select *M*, with *N* electrodes being Electrodes 2 and 3, and collect voltage $V_{MN1}$ and $V_{MN3}$ between MN before sending it to the operator control unit.
5. After receiving $V_{Rx2}$, the user operates the unit in $R_{x}$ to obtain the output current *I* from the power supply. Then, the apparent resistivity collected by Data Acquisition Unit 2 is calculated using Equation (1), and the apparent resistivity collected by Data Acquisition Units 1 and 3 is calculated using Equation (2).

In the described data exchange process, if the operator control unit fails to receive feedback data within 5 s, the collection is deemed incorrect and the process restarts from Step 1. During the collection process, the operator control unit displays the acquired data shown in display interface, as illustrated in Figure 7b.

The PC operation interface of the operator control unit, designed using QT, is depicted in Figure 7b, with the operation process shown in Figure 7a. Upon launching the interface, the first step involves opening the serial port to enable data interaction with the ZigBee module. Subsequently, the user adds grouping information for the data collection unit, such as the number of electrodes, electrode spacing of *a*, and line spacing of *d*, based on the actual power-on sequence and wiring configuration. The user control unit, as designed in this article, allows setting the interval and frequency for continuous data collection. It automatically issues collection commands based on these settings, eliminating the need for manual repetition and thereby enhancing efficiency.

The ZigBee module connects to the PC using a USB to TTL converter, with the serial port configured via the operation interface. With this setup, the PC sends data to the ZigBee module over a serial channel. Consequently, the PC, through this module, becomes part of the ZigBee network, acting as the the operator control unit and facilitating human–machine interaction.

**Figure 8:**
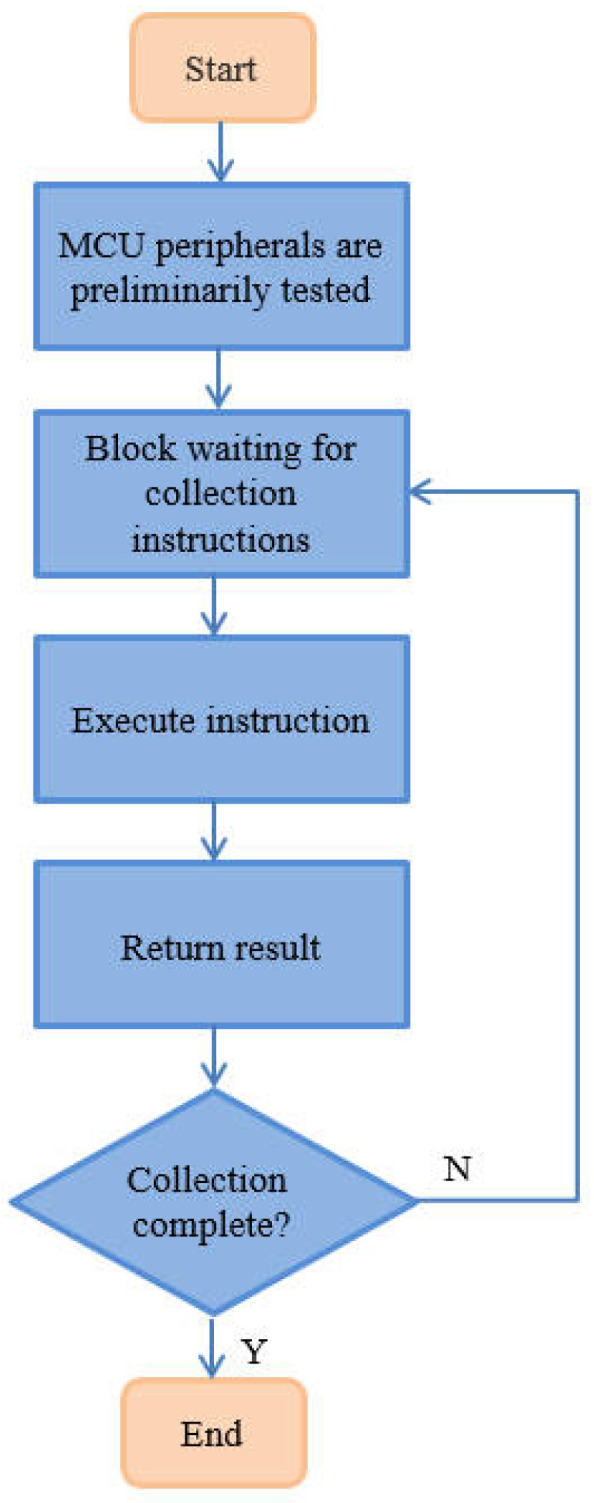
MCU workflow of data collection unit.

For the MCU of the data collection unit, the program flow is illustrated in Figure 8. After power on, STM32 initializes the IO, serial port, and other peripherals used. After receiving instructions, the following operations are mainly performed:

1. Following the provided electrode number, the MCU calculates a 60-bit binary code for the 60 relays and sends the control signal to the 74HC595 via IO, enabling the relays associated with electrodes *A*, *M*, *N*, and *B* for acquisition purposes.
2. The MCU reads the voltage values collected by the IN1, IN2, and IN3 channels of ADS131E08 through SPI communication. These three channels are for the MN electrode voltage $V_{MN}$, sampling resistor voltage $V_{Rx}$, and discharge power supply voltage $V_{B}$.
3. $V_{MN}$ and the output current *I* are measured from the battery pack using the voltage divider, then the data are transferred via the serial port to the ZigBee module, which forwards it to the operator control unit.

**Figure 9:**
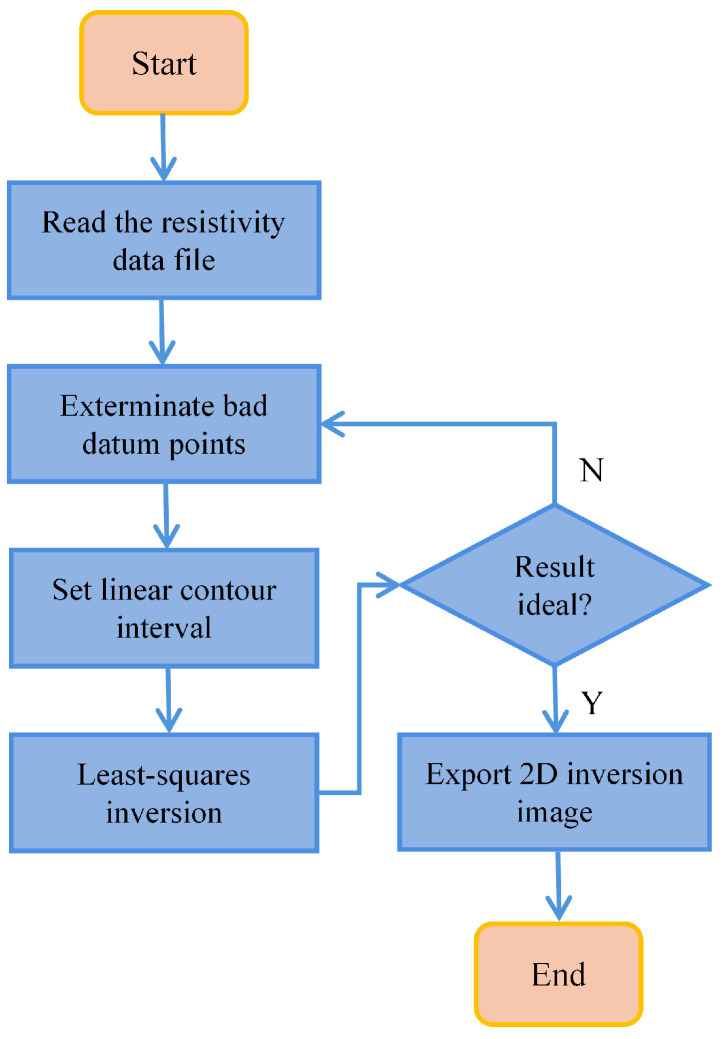
Flowchart of single profile inversion.

After completing data acquisition, the measurement data of each profile can be exported through the user control interface and processed individually using 2D inversion. The inversion flowchart is shown in Figure 9.

Each profile’s data are inverted using the RES2DINV software (version 3.54u) with the least squares algorithm, setting the number of iterations to 5 and the contour interval to 10, while ensuring that the root mean square (RMS) error remains below 5% to guarantee accuracy. The 2D inversion results of multiple profiles can then be integrated to form a 3D composite visualization through GoCAD, enabling a spatially intuitive display of the grout diffusion process.

## 6. Test and Result

### 6.1. System Acquisition Performance Test

**Figure 10:**
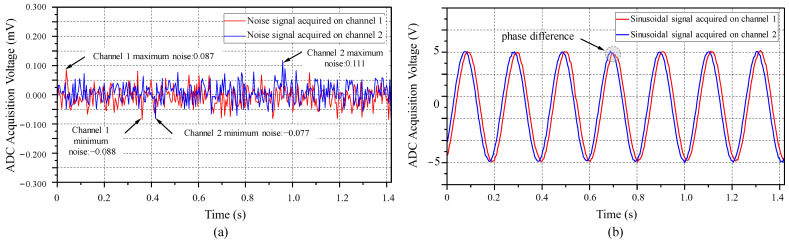
Analysis of the steady-state performance of the data collection unit. (**a**) Channel noise acquisition test. (**b**) Sinusoidal signal input acquisition test.

The accuracy of the voltage measurement of the data acquisition unit is crucial and directly affects the grouting detection effect. Therefore, the system acquisition performance needs to be tested. The input units of the two differential input channels of the ADS131E08 were short-circuited to obtain the channel noise as shown in Figure 10a. The ADS131E08 chip was set to a sampling rate of 1 kHz. In the experiment, 1400 data points were sampled for each channel, and by subtracting the maximum value from the minimum value, the equivalent input noise (EIN) was obtained as 175 μV for Channel 1 and 188 μV for Channel 2. A sine wave voltage with a peak-to-peak value of 10 V was input into the two channels, and the result is shown in Figure 10b, with a phase difference of 3.6° between the two channels. Compared with the generated standard sinusoidal waveform, the average errors of the sinusoidal waveform data collected by the channels were calculated to be, respectively, 0.004514 V and 0.00419 V. During data collection, the ADC device uses two channels: $V_{MN}$ (the voltage of the acquisition channel) and $V_{Iout}$ (the voltage across the sampling resistor to obtain the discharge current). We performed SNR measurements on these two channels, which yielded results of 98.76 dB and 97.66 dB, respectively.

### 6.2. Actual Measurement Experiment

**Figure 11:**
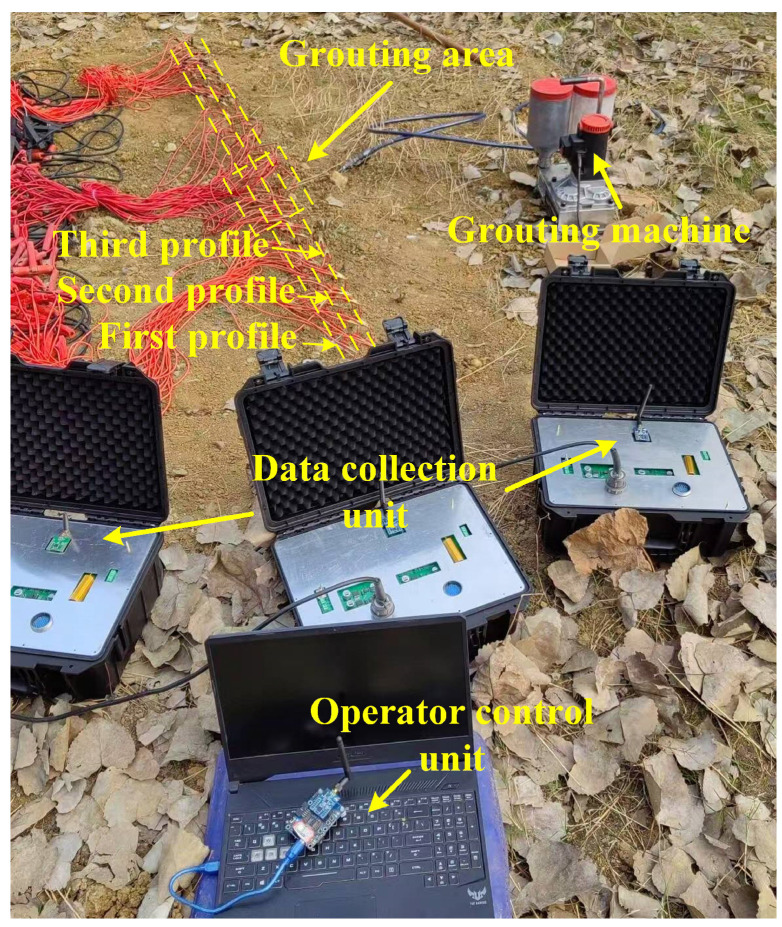
Experimental testing of three parallel lines collecting grouting sites.

In order to test the actual functional effectiveness of the system, a grouted area was selected outdoors for monitoring experiments. The slurry injection area was a rectangular area of 40 cm length, 20 cm width, and 20 cm height, modified to simulate a pebble layer, resulting in a higher apparent resistivity than the surrounding area. When slurry is injected into the area, the low resistivity of the slurry causes the apparent resistivity of the area to decrease. The apparent resistivity of the injected region was monitored in real time by the system to verify the feasibility of the system for real-time monitoring of the spreading of the slurry and the effect of the slurry injection. The experimental setup includes an operation control unit, acquisition units, a grouting machine, electrodes, and measuring lines. The acquisition unit, equipped with a ZigBee node, facilitates wireless communication with the operation control unit. The measuring lines are arranged in the grouting area and the electrodes were connected to the measuring lines. The acquisition unit connects the measurement lines to control the electrodes and collect data. As shown in Figure 11, the arrangement consists of three measuring lines, each 4 cm apart. The electrodes are spaced 8 cm apart and are made of copper wire with a diameter of 1.78 mm. Each measuring line is connected to 30 electrodes. The second measuring line passes through the center of the grouting area. The center of the grouting area is approximately 1.25 m from the first electrode and 2.32 m from the last electrode. The grouting machine is used to perform the grouting at a pressure of 3.45 MPa and the grouting time is 20 min. The grouting material used is a cement-based slurry consisting of Ordinary Portland Cement, water, and graphite powder mixed in a ratio of 5:2.64:1. Cement is a typical low-resistivity grouting material, and the addition of graphite powder enhances the electrical conductivity and fluidity of the slurry, facilitating the measurement of electrical parameters.

**Figure 12:**
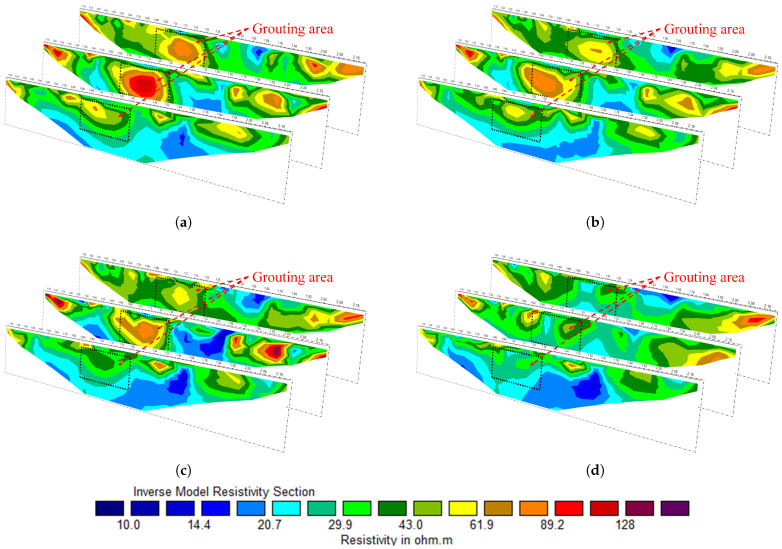
Actual inversion results. (**a**) Apparent resistivity distribution in the subsurface before grouting. (**b**) Apparent resistivity distribution after 5 min of grouting in the subsurface. (**c**) Apparent resistivity distribution after 10 min of grouting in the subsurface. (**d**) Apparent resistivity distribution 20 min after grouting.

Data were acquired from the area before grouting and inversion analysis was performed, yielding inversion diagrams for three profiles; the result is shown in Figure 12a. In the simulated pebble layer area, the apparent resistivity was significantly higher than that of the surrounding area, with a maximum value of about 120 Ω·m, while the surrounding area ranged from 10 to 50 Ω·m. After 5 min of grouting, data were acquired again for inversion analysis, as shown in Figure 12b. At this time, the apparent resistivity of the grouted area decreased, with the maximum resistivity dropping to about 90 Ω·m. This decrease is consistent with the expected reduction in apparent resistivity when the slurry fills the voids. The process was continued, and after 10 min of slurry injection, the result was again taken and inverted as shown in Figure 12c. The high-resistivity region gradually narrowed and the apparent resistivity of the grouted region further decreased. Finally, after 20 min of grouting, the high-apparent-resistivity region within the original grouted area disappeared, as shown in Figure 12d. During the experiment, we can monitor the spatial change in the slurry in the grouting region and the grouting effect through the real-time apparent resistivity inversion of the system, which indicates that the real-time monitoring system developed in this study can be effectively used to monitor and control the grouting process. However, due to the limited size of the grouting area in the experiment, the slurry diffusion boundary could not be analyzed. In practical grouting applications, the grouting area is usually larger and the grouting time is longer. Therefore, further practical testing is required to fully assess the system’s capabilities.

### 6.3. Construction Field Testing

**Figure 13:**
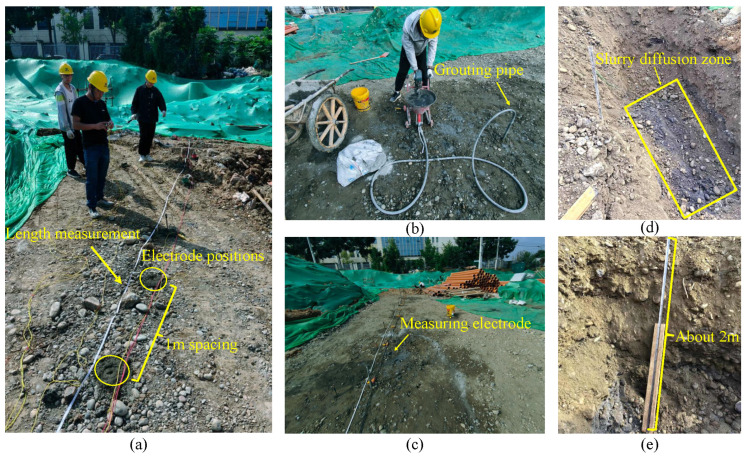
Field grouting experiment. (**a**) Measurement before grouting. (**b**) Grouting process with grouting pipe. (**c**) Measurement after grouting. (**d**) Excavation verification. (**e**) Depth verification.

To verify the feasibility of the system in practical application scenarios, a test was conducted at a road construction site in Chengdu, Sichuan Province, China (E104.23°, N30.71°). The site exhibited complex geological conditions dominated by poorly sorted cobblestone strata. Uneven settlement and ground subsidence occurred during the construction process, which required foundation improvement by grouting and filling. Three measuring lines were deployed at the grouting site for data collection in the grouting area, with each line being 30 m long (30 electrodes spaced 1 m apart). The testing process is shown in Figure 13. The background voltage values were first measured using our system, followed by inversion imaging to determine the collapse or void locations. Grouting was then performed in the anomalous area, approximately at the 10th and 20th electrode positions along the measuring line. The system measured the apparent resistivity before grouting, 2 h after grouting and 24 h after grouting, respectively.

**Figure 14:**
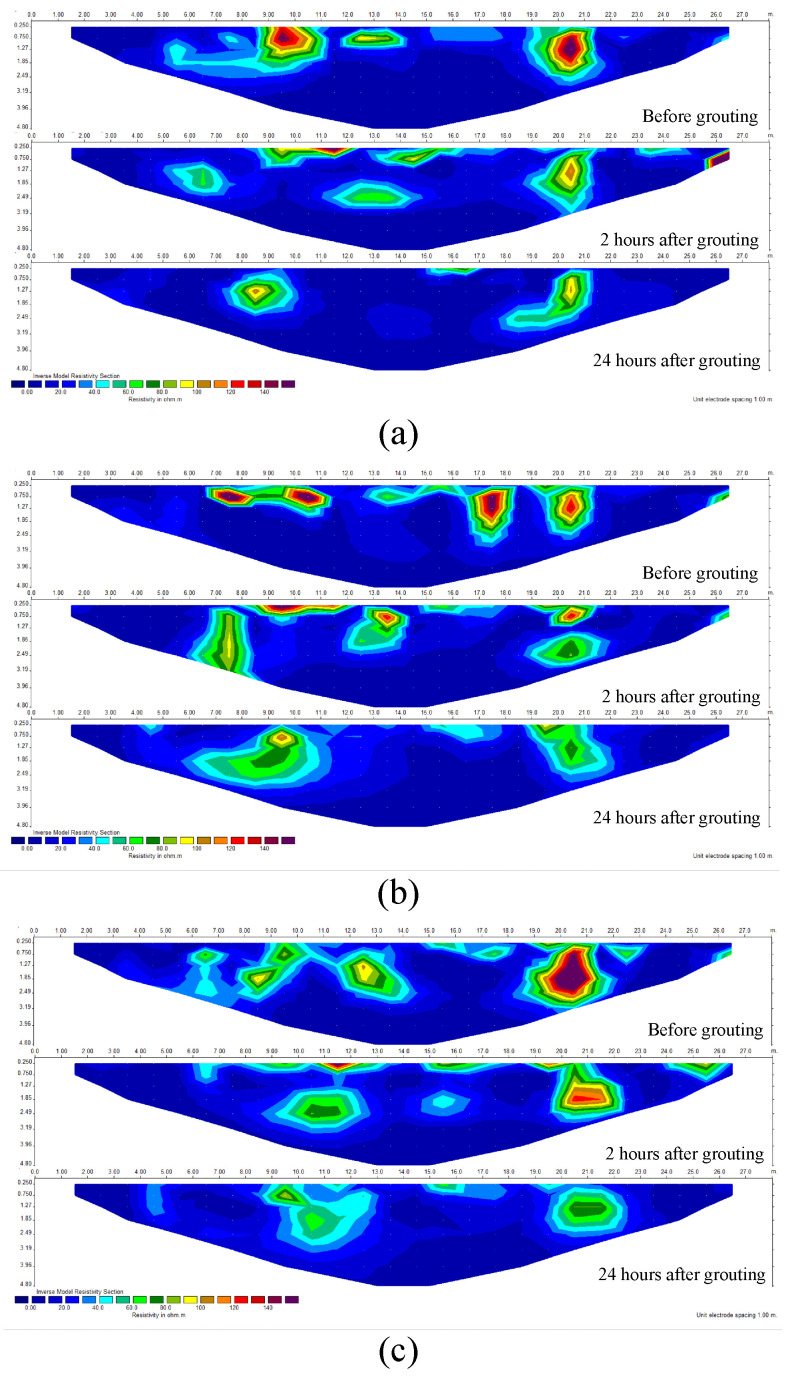
Actual engineering measurement inversion diagrams. (**a**) Inversion of the first measuring line. (**b**) Inversion of the second measuring line. (**c**) Inversion of the third measuring line.

The apparent resistivity results of the site before grouting, 2 h after grouting, and 24 h after grouting are shown in Figure 14. Comparing the anomaly inversion results before and after grouting, combined with excavation verification (as shown in Figure 13d,e), it can be concluded that the system can effectively display the grouting filling range and detect the slurry diffusion. The high-resistance anomaly range is significantly reduced after grouting, indicating that the slurry has effectively filled the collapsed or fissure areas. Additionally, while the anomaly locations along the three measuring lines are generally consistent, the anomaly shapes vary, which is related to geological heterogeneity.

### 6.4. System Stability and Adaptability Testing

To evaluate the long-term performance of the system, we conducted tests in both road construction and building construction environments. In each environment, the system was deployed at the same location, and apparent resistivity measurement was performed weekly. A 30 m long measuring line was laid out on site, with 30 electrodes placed 1 m apart. A total of 135 measurement points were collected per measurement. The measurements continued for 8 weeks, providing 8 sets of data for each point. To assess the long-term stability of the system, we used the Relative Standard Deviation (RSD) as an evaluation metric, calculated for each measurement point using Equation (7):

(7) $$RSD=\frac{s}{\bar{x}}\times 100\%$$

In the formula, $\bar{x}$ is the mean of a given measurement point and *s* is the standard deviation, representing the degree of data dispersion and fluctuation. The standard deviation is calculated using Equation (8):

(8) $$s=\sqrt{\frac{1}{n-1}\sum_{i=1}^{n}{\left(x_{i}-\bar{x}\right)}^{2}}$$

where *n* is the number of measurements, $x_{i}$ is the apparent resistivity value, and $\bar{x}$ is the mean value for the measurement point.

While the standard deviation can also measure data dispersion, its value is directly influenced by the magnitude of the data. In areas with significant differences in apparent resistivity, the standard deviation may not accurately reflect the system’s true stability. However, RSD divides the standard deviation by the mean, providing a clearer indication of relative data fluctuation. A lower RSD value indicates lower data dispersion and higher system stability. Additionally, to assess the system’s adaptability under different environmental conditions, we compared the consistency of measurement data from both environments. By analyzing the proportion of measurement points in the RSD, the environmental impact on the system can be inferred.

**Figure 15:**
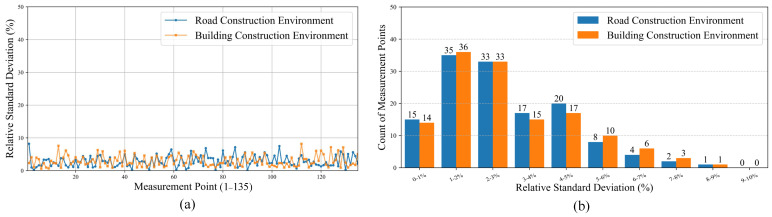
Long-term stability and environmental adaptability test of the system. (**a**) The RSD of measurement points in road and building construction environments. (**b**) The distribution of measurement points within different RSD ranges.

The test results are shown in Figure 15a. In the road construction environment, the RSD values range from 0.11% to 8.21%, with a mean of 2.86% and a variance of 2.94%. In the building construction environment, the RSD values range from 0.59% to 8.19%, with a mean of 2.92% and a variance of 2.97%. Considering the heterogeneity of the geology and the temporal variation of the environment, an RSD value within 5% indicates good measurement stability of the equipment. In both environments, the mean RSD values are below 5%, indicating that the data fluctuations collected by the equipment are small, and the system demonstrates good long-term stability. Additionally, the mean RSD values and variances of both environments are similar. As shown in Figure 15b, the proportion of measurement data within different RSD values is similar in both environments, demonstrating high consistency. This suggests that the environmental impact on the equipment is minimal, and the system exhibits good environmental adaptability.

### 6.5. Network Performance Measurement

**Figure 16:**
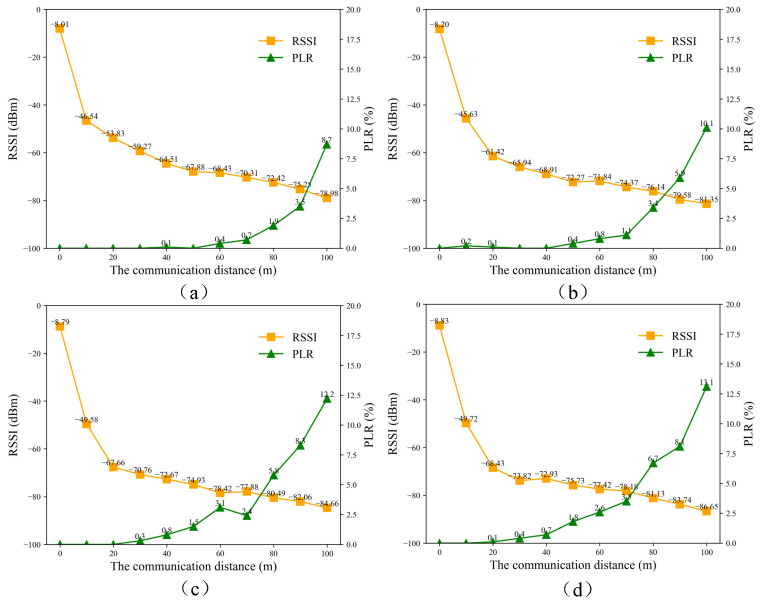
Communication quality test results of the ZigBee network in different scenarios. (**a**) Enclosed corridor. (**b**) Urban open area. (**c**) Complex area. (**d**) Construction site.

The ZigBee network is used in the system for data transmission and expanding the network of acquisition unit nodes, so it is crucial to verify the performance of the ZigBee network. We tested the ZigBee packet loss rate (PLR) from 0 to 100 m in different environments. In this experiment, two ZigBee nodes were deployed, one as a transmitter and the other as a receiver. The receiver was in a fixed position, while the transmitter was mobile. The transmitter operated at a power level of 4 dBm and sent 1000 packets at 0.5 s intervals. The receiver recorded the number of successfully received packets, the number of lost packets, and the RSSI value. Packet loss was identified by detecting jumps in the sequence number, and erroneous packets were categorized as lost. The experiments were conducted in four scenarios: closed corridor, open urban area, complex area, and a grouting construction site. The closed corridor simulated a tunnel-like or narrow environment with dimensions of 2.84 m in height and 2.74 m in width. The urban open area simulated a flat and obstacle-free urban grouting site. The complex area simulated an environment with obstacles such as trees and undulating terrain. The construction site provided a practical grouting environment to evaluate the feasibility of the ZigBee network. The measurement results of these scenarios are shown in Figure 16. In the tests, a PLR of less than 2% is considered to be an indicator of good communication quality. In all four scenarios, the ZigBee network performed well in the transmission range of 0 to 50 m. However, between 50 and 100 m, the PLR increased rapidly and the communication quality decreased. The experimental results of RSSI and PLR showed that the communication quality at the same distance was best in the closed corridor, followed by the open area, then the complex area, and finally the construction site. Compared to the open area, the complex area and construction site had lower values of RSSI and higher values of PLR, which were due to the effect of terrain undulation and obstacles, leading to unstable communication quality. In this case, ZigBee routing can be used to extend the communication link and ensure communication stability. It is worth noting that in typical grouting engineering applications, the distance between the operation control unit and the acquisition unit is generally short, and a communication range of 50 m is sufficient for the majority of engineering scenarios. In addition, to verify the ability of the ZigBee router to relay signals and maintain network stability, we conducted experiments using a ZigBee coordinator, a router, and an end device. These three nodes were arranged in a straight line with equal spacing. The end device sent 5000 packets at 1-second intervals, each with a payload of 40 bytes. The test scenarios were consistent with those described earlier. As shown in Table 2, the packet loss rates were less than 2% in all three test scenarios when the distances did not exceed 50 m, which confirms the reliability and stability of the network under these conditions.

The ZigBee network adopts a multi-hop communication mechanism. When the distance between two nodes exceeds the transmission range, the signal can be transmitted through relay. By reasonably arranging ZigBee routers, communication links can be established to ensure the continuity of data transmission. In environments with physical barriers or irregular terrain, a mesh network topology is used to optimize the layout of routers and end devices, and additional routing nodes are added as needed. In addition, redundant ZigBee routers can be deployed to enhance the robustness of the network in case of router failure.

## 7. Discussion

Previous grouting evaluation methods have achieved acceptable results but are fraught with significant drawbacks. For instance, invasive methods such as the inspection hole method and excavation sampling cause damage to the grouted area and are incapable of real-time monitoring of slurry diffusion and grouting effectiveness. Meanwhile, non-invasive methods like ground-penetrating radar are limited in their detection depth. Traditional direct current methods, which rely on sequential measurements, require the repeated removal and reinsertion of electrodes for data acquisition across different profiles. This process not only increases operational complexity but also introduces potential inaccuracies and cumulative errors due to variations in contact resistance. Therefore, this paper proposes a ZigBee-based grout monitoring sensor system to address these issues.

To further clarify the advantages of the proposed system, a comparison with commercial alternatives such as Distributed Fiber Optic Sensing (DFOS) is provided. While DFOS has shown feasibility in recent experimental studies for monitoring slurry diffusion [51,52], it faces limitations in real projects: (1) Complex and time-consuming fiber deployment, especially in large-scale sites, with high demodulation equipment costs; (2) Large data volume makes real-time processing difficult [53]; (3) Resolution is limited by fixed fiber layouts and grid spacing, reducing adaptability.

In contrast, our system only requires surface electrode placement, allowing for faster and simpler deployment. Its resolution is easily adjustable by modifying electrode density and spacing. Moreover, the system is low-cost, requires no consumable materials such as optical fibers, and supports real-time, non-invasive monitoring with minimal disruption to construction activities. Importantly, the system is based on the well-established DC resistivity method, widely used in geological and grouting-related applications, making it easier for industry adoption and offering strong potential for standardization based on its advantages.

The key innovations of our work can be summarized in the following aspects:

1. A new 60-channel integrated system is developed. This highly integrated design eliminates the labor cost of manually switching electrodes, significantly reduces operating time and improves measurement efficiency. Moreover, the method prearranges the electrodes in the measurement area, removing the need for repeated insertion and removal, thereby eliminating measurement errors associated with electrode reinsertion. The system employs multi-channel synchronized measurement to ensure the independence of data acquisition for each profile, which effectively mitigates the cumulative error and discharge effect, ensuring the accuracy and reliability of the measurement.
2. The system’s acquisition unit can sense discharge voltage and perform data acquisition for three profiles simultaneously to conduct a three-dimensional inversion, providing a more detailed and intuitive way to monitor the diffusion of the slurry. Compared with previous measurement methods, the system not only improves the efficiency of data collection but also updates the inversion results in real time to reflect the time-varying characteristics of the slurry, which helps to provide quick feedback on the grout performance and slurry diffusion results during construction.
3. The new system is equipped with a ZigBee module for wireless data transmission, allowing the control unit to receive and process the collected data in real time. This ensures efficient monitoring of the grouting process. In addition, the design supports the addition of more acquisition units to form an extensive network, expanding the measurement range, and enhancing scalability.

However, this method has both applicability and limitations. As the depth measured by the method is proportional to the length of the measuring line, the system is suitable for large-scale and open-area grouting works, such as the reinforcement of urban foundations, the backfilling of air-mined areas in mining works, and the hardening of coastal strata. However, the measurement line cannot be arranged in a curved manner and the detection depth is limited by the length of the line, so it is not suitable for narrow or curved spaces such as tunnels where deploying the measurement line is challenging due to spatial limitations. Additionally, the ZigBee network used in the system has a limited communication range. Extending this range requires the addition of extra ZigBee routers, which increases costs. Also, rainwater infiltration and terrain undulations can affect the underground current distribution and reduce the measurement accuracy. To reduce the disturbances, measurements should be performed under dry and flat terrain conditions. In the future, we plan to consider more aspects of system improvement, especially improving the circuitry to further increase the resolution and adaptability of the monitoring system, as well as developing a wall-mounted design for confined spaces and improved algorithms to minimize the effects of terrain variations, thereby improving the system’s robustness and practical performance.

## 8. Conclusions

The article introduces a sensor system for real-time monitoring of grout diffusion. The system consists of a user control unit, a ZigBee module, and one or more data acquisition units. We conducted comprehensive tests, including acquisition performance evaluation, practical validation, on-site testing at construction sites, and performance assessment of the ZigBee sensor network. Additionally, we developed a communication program based on the ZigBee application layer, enabling the control unit to connect with multiple data acquisition units and form a scalable data acquisition network for various grouting project sizes.

The system acquisition performance test showed that the channel noise level of the data acquisition unit was extremely low, with equivalent input noise (EIN) values of 175 μV and 188 μV. When a sine wave with a peak-to-peak value of 10 V was input, the average errors were only 4.51 mV and 4.19 mV, respectively, verifying the system’s accurate acquisition capability. In the actual measurement experiment, the system was able to monitor the changes in apparent resistivity in the simulated grouting area in real time, with the grout diffusion process clearly visualized. In the construction site test, the system successfully monitored the changes in apparent resistivity before and after grouting at a road construction site. The high-resistance anomaly range significantly decreased after grouting, indicating that the grout effectively filled the collapse or fissure areas, demonstrating the system’s feasibility in engineering applications. Moreover, the system stability test showed that in road and building construction environments, the mean relative standard deviation (RSD) values of the system were 2.86% and 2.92%, respectively, both below 5%, indicating good long-term stability and environmental adaptability. In the ZigBee network performance test, three engineering environments were simulated and tested on-site. The results showed that within a transmission range of 0 to 50 m, the packet loss rate (PLR) of the ZigBee network was below 2%, indicating good and reliable communication quality.

Through these experiments, the system demonstrated its ability to collect real-time data from multiple profiles and invert apparent resistivity to visualize slurry spread and grouting diffusion effect. This provides a valuable reference for grouting projects. However, we also identified limitations of the current system. Future work will focus on improving the data acquisition unit’s circuitry to accommodate higher discharge voltages, expanding the real-time monitoring area, enhancing accuracy, and developing a wall-mounted design for narrow spaces. Furthermore, we plan to improve the algorithm to minimize the influence of terrain variations, thereby improving the system’s robustness and practicality.

## Figures and Tables

**Table 1:** Comparison of Communication Standards.

Standard	Bluetooth	ZigBee	Wi-Fi	LoRa	NB-IoT
Frequency band	2.4 GHz	868/915 MHz; 2.4 GHz	2.4 GHz; 5 GHz	433, 780, 868, 915 MHz	400, 900, 2700 MHz
Max signal rate	1 Mb/s	250 Kb/s	54 Mb/s	50 Kb/s	253.6 Kb/s
Nominal range	10 m	10–150 m	100 m	5 km (urban), 18 km (rural)	10 km (rural)
Nominal TX power	0–10 dBm	(−28)–4.5 dBm	15–20 dBm	10–20 dBm	23 dBm

**Table 2:** Packet loss rate of ZigBee node spacing.

Scenario	ZigBee Node Spacing				
	10 m	20 m	30 m	40 m	50 m
Corridor	0.00%	0.02%	0.10%	0.22%	0.76%
Open Area	0.00%	0.06%	0.38%	0.80%	1.36%
Complex Area	0.00%	0.24%	0.66%	1.96%	1.62%
Construction site	0.00%	0.08%	0.42%	1.24%	1.84%

## Data Availability

Data and source code used in the paper can be accessed by contacting the authors.
